# Creatine Enhances Mitochondrial-Mediated Oligodendrocyte Survival After Demyelinating Injury

**DOI:** 10.1523/JNEUROSCI.1941-16.2016

**Published:** 2017-02-08

**Authors:** Kelly A. Chamberlain, Kristen S. Chapey, Sonia E. Nanescu, Jeffrey K. Huang

**Affiliations:** ^1^Department of Biology and; ^2^Interdisciplinary Program in Neuroscience, Georgetown University, Washington, District of Columbia 20057

**Keywords:** apoptosis, creatine, demyelination, guanidinoacetate-methyltransferase, oligodendrocyte, regeneration

## Abstract

Chronic oligodendrocyte loss, which occurs in the demyelinating disorder multiple sclerosis (MS), contributes to axonal dysfunction and neurodegeneration. Current therapies are able to reduce MS severity, but do not prevent transition into the progressive phase of the disease, which is characterized by chronic neurodegeneration. Therefore, pharmacological compounds that promote oligodendrocyte survival could be beneficial for neuroprotection in MS. Here, we investigated the role of creatine, an organic acid involved in adenosine triphosphate (ATP) buffering, in oligodendrocyte function. We found that creatine increased mitochondrial ATP production directly in oligodendrocyte lineage cell cultures and exerted robust protection on oligodendrocytes by preventing cell death in both naive and lipopolysaccharide-treated mixed glia. Moreover, lysolecithin-mediated demyelination in mice deficient in the creatine-synthesizing enzyme guanidinoacetate-methyltransferase (*Gamt*) did not affect oligodendrocyte precursor cell recruitment, but resulted in exacerbated apoptosis of regenerated oligodendrocytes in central nervous system (CNS) lesions. Remarkably, creatine administration into *Gamt*-deficient and wild-type mice with demyelinating injury reduced oligodendrocyte apoptosis, thereby increasing oligodendrocyte density and myelin basic protein staining in CNS lesions. We found that creatine did not affect the recruitment of macrophages/microglia into lesions, suggesting that creatine affects oligodendrocyte survival independently of inflammation. Together, our results demonstrate a novel function for creatine in promoting oligodendrocyte viability during CNS remyelination.

**SIGNIFICANCE STATEMENT** We report that creatine enhances oligodendrocyte mitochondrial function and protects against caspase-dependent oligodendrocyte apoptosis during CNS remyelination. This work has important implications for the development of therapeutic targets for diseases characterized by oligodendrocyte death, including multiple sclerosis.

## Introduction

Oligodendrocytes are glial cells of the CNS that myelinate axons to promote saltatory conduction and provide neurons with energy metabolites, including lactate (Fünfschilling et al., 2012; Lee et al., 2012). Oligodendrocyte and myelin loss is a hallmark feature of the chronic inflammatory disease multiple sclerosis (MS) (Wolswijk, 1998; Compston and Coles, 2008). Although existing MS therapies are able to reduce disease severity, they do not prevent progression into the chronic neurodegenerative phase of the disease (Rice, 2014). Myelinating oligodendrocytes regenerate spontaneously after demyelination in the early phase of MS because of the availability of endogenous oligodendrocyte precursor cells (OPCs) in the CNS (Franklin and Gallo, 2014). However, this process becomes increasingly difficult with disease progression and results in progressive axonal loss and the accumulation of clinical disability (Franklin, 2002; Haines, Inglese, and Casaccia-Bonnefil, 2009; Fitzner and Simons, 2010; Dutta and Trapp, 2011). Why remyelination ultimately fails in MS is not well understood. One possibility is that oligodendrocytes do not survive well under the inflammatory environment in MS. For example, oligodendrocytes are highly vulnerable to hypoxic-ischemic injury (Ness et al., 2001; McIver et al., 2010; Jablonska et al., 2012), a feature of MS lesions (Lassmann, 2016), and readily undergo inflammation-mediated cell death (Vartanian et al., 1995; Akassoglou et al., 1998; Baerwald and Popko, 1998).

Previous studies have demonstrated that oligodendrocyte survival can be achieved in murine autoimmune-mediated models of demyelination by overexpressing the anti-apoptotic protein p53 (Hisahara et al., 2000), by enhancing the integrated stress response (Lin et al., 2007; Way et al., 2015), or by deleting FADD (Mc Guire et al., 2010), a receptor adaptor protein involved in apoptotic initiation. Moreover, mice with enhanced oligodendrocyte survival also displayed reduced disease severity. These studies suggest that enhancing oligodendrocyte survival would be beneficial as a treatment strategy in MS.

One candidate for promoting oligodendrocyte survival is creatine, a cytoprotective organic acid (Matthews et al., 1998, 1999; Klivenyi et al., 1999; Andres et al., 2005) that has been shown to regulate neuronal mitochondrial activity (Lee and Peng, 2008) and protect against oxidative damage (Sestili et al., 2006; Hosamani et al., 2010; Berti et al., 2012; Saraiva et al., 2012). Creatine is hypothesized to function as an intracellular ATP buffer because it is phosphorylated near sites of ATP production by mitochondrial creatine kinase (MtCK) to generate phosphocreatine, which is then reversibly dephosphorylated by cytoplasmic creatine kinases for rapid ATP regeneration near sites of high utilization (Wyss and Kaddurah-Daouk, 2000). Intriguingly, oligodendrocytes have the highest capacity for the synthesis and utilization of creatine in the CNS (Manos et al., 1991; Molloy et al., 1992; Braissant et al., 2001; Tachikawa et al., 2004; Cahoy et al., 2008; Zhang et al., 2014). In addition, inborn errors of creatine metabolism frequently present with delayed myelination, as well as severe mental retardation, autistic-like behavior, motor disorder, and speech delay, suggesting a crucial role for creatine during brain development (Anselm et al., 2006; Barkovich, 2011). Despite its clinical relevance, the precise role of creatine in oligodendrocytes remains poorly understood.

Here, we investigated the role of creatine in oligodendrocyte function. We demonstrate that creatine increases oligodendrocyte mitochondrial ATP production directly and promotes oligodendrocyte survival under inflammatory conditions *in vitro* and after focal demyelination *in vivo* in mice. Moreover, we show that creatine does not affect the distribution of macrophages/microglia in demyelinated lesions, suggesting that the protective effect of creatine occurs independently of inflammatory modulation. These results suggest that the therapeutic administration of creatine may promote oligodendrocyte survival in MS.

## Materials and Methods

### 

#### 

##### Mice.

C57BL/6J mice were obtained from The Jackson Laboratory and provided food and water *ad libitum. Gamt*^−/+^ mice were a kind gift from Dr. Dirk Isbrandt (University of Cologne). All experiments were performed according to protocols approved by the Institutional Animal Care and Use Committee at Georgetown University.

##### Chemicals and antibodies.

Lipopolysaccharide (LPS; *Escherichia coli* 0111:B4) was obtained from InvivoGen. Creatine monohydrate (C3630), 3-guanidinopropionoic acid (GPA; G6878), and carbonyl cyanide 4-(trifluoromethoxy)phenylhydrazone (FCCP; 370–86-5) were obtained from Sigma-Aldrich. MitoTracker Red FM (M22425), tetramethylrhodamine, ethyl ester (TMRE; T669), and propidium iodide (PI) (P3566) were obtained from Thermo Fisher Scientific. Primary antibodies for immunohistochemistry (IHC) were as follows: rat anti-CD11b (1:100; AbD Serotec), rabbit anti-cleaved caspase-3 (1:100; Cell Signaling Technology), rabbit anti-Olig2 (1:300; Millipore), rat anti-myelin basic protein (MBP, 1:200; AbD Serotec), mouse anti-CC1 (1:300; Millipore), and mouse anti-Nkx2.2 (1:100; Developmental Studies Hybridoma Bank). Primary antibodies for immunocytochemistry (ICC) were as follows: rabbit anti-Olig2 (1:500; Millipore), rat anti-MBP (1:500; AbD Serotec), anti-GFAP (1:500; Sigma-Aldrich), mouse anti-CC1 (1:200; Millipore), and anti-PDGFRα (1:300; BD PharMingen). Alexa Fluor 488 or 594 secondary antibodies (Thermo Fisher Scientific) were used at a concentration of 1:500. To label nuclei, cell and tissues were labeled with 1 μg/ml Hoechst in PBS for 5 min at room temperature (RT) (33342; Thermo Fisher Scientific).

##### Cell cultures.

Mixed glia cultures were prepared from postnatal day 3 (P3) to P5 mouse cortices as described previously (Daniele et al., 2014) and maintained in DMEM-F12 containing 10% FBS, 1% penicillin-streptomycin (P/S), 1% Fungizone (F/Z), and 1% GlutaMAX for 2 weeks. Mixed glia cultures were switched to a serum-free medium for 24 h before initiating treatments. Primary OPCs were established by sequential shaking of confluent mixed glia cultures as described previously (Daniele et al., 2014). Primary oligodendrocyte lineage cell cultures were obtained from P3–P5 mouse cortices using magnetic activated cell sorting (MACS) with the Neural Tissue Dissociation Kit (130-092-628) and anti-O4 microbeads (130-096-670) according to the manufacturer's protocol (Miltenyi). Primary oligodendrocyte lineage cells were expanded in growth medium (DMEM-F12 with N2, B27, P/S, F/Z, BSA, FGF and PDGF) and then differentiated using defined medium (DMEM-F12, N2, B27, P/S, F/Z, insulin, and T3) as described previously (Dincman et al., 2012). OLN-93 cells (Richter-Landsberg and Heinrich, 1996), originally from Dr. Christiane Richter-Landsberg (University of Oldenburg), were kindly provided by Dr. Wendy Macklin (University of Colorado) and maintained in DMEM containing 10% FBS, 1% P/S, 1% F/Z, and 2 mm glutamine. All cultures were treated with 100 μm creatine, 100 μm 3-guanidinopropionic acid, and/or 1 μg/ml LPS for 24–48 h.

##### Spinal cord demyelination.

Focal demyelination was induced by injection of 1.0% lysolecithin (Sigma-Aldrich) diluted in sterile PBS into the spinal cord ventral funiculus of 9- to 12-week-old mice. In all mice, 1× PBS or creatine monohydrate (25 ng) was coinjected along with 1.0% lysolecithin. The animals were killed for analysis at 5, 10, or 20 d after surgery. Both male and female mice were used for surgeries because no differences were observed between sexes.

##### Immunochemistry.

Mice were perfusion fixed with 4% (w/v) PFA (Sigma-Aldrich) in PBS. Spinal cord tissue was dissected and postfixed for 45 min in 4% PFA at RT. Tissue was cryoprotected in 20% (w/v) sucrose (Sigma-Aldrich) in PBS at 4°C overnight before freezing in optimal cutting temperature medium on the surface of dry ice. Then, 12 μm frozen spinal cord sections were collected on SuperFrostPlus slides (Stellar Scientific) using a cryostat and dried for 30 min before storage at −80°C. For *in vitro* experiments, cells were fixed with 4% (w/v) PFA and 120 mm sucrose for 20 min and then washed with PBS. Sections/cells were then incubated in blocking solution (0.01% v/v Triton X-100 and 5% goat serum in PBS) for 1 h at RT. Primary and secondary antibodies were diluted in PBS containing 0.01% (v/v) Triton X-100 and 1% (w/v) BSA and applied to sections/cells overnight at 4°C. TBS was substituted for PBS when immunolabeling tissue sections. For detection of Nkx2.2 and CC1, mouse-on-mouse antigen retrieval was performed before immunohistochemistry according to the manufacturer's instructions (M.O.M. kit; Vector Laboratories).

##### Western blot.

Cerebellum was dissected from mice at various postnatal time points. Proteins were harvested in lysate buffer (150 mm sodium chloride, 1% Triton X-100, and 50 mm Tris, pH 8.0), separated by SDS-PAGE, and immunoblotted using the following antibodies: mouse anti-VDAC (1:50; Abcam) and rabbit anti-Tom20 (1:50; Santa Cruz Biotechnology). Proteins were detected using horseradish peroxidase-conjugated secondary antibodies and Pierce ECL Western blotting substrate. Protein expression was quantified by densitometry and represented as average protein expression normalized to β-Actin loading control.

##### Imaging and cell counting.

For quantification of immunostaining, a blinded investigator used the ImageJ cell counter to count cells manually from low-magnification (10–20×) images. For mixed glia cultures, data are represented as proportion of total Olig2^+^ cells to control for the heterogeneous density and growth of mixed glia cultures. For analysis of normal appearing white matter (NAWM) in uninjured spinal cord, cells were counted in spinal cord tissue directly adjacent to ventral lesions. For analysis of focal demyelinating spinal cord injury, cells were counted only in the lesioned area. Lesions were identified by abnormally high cell density in the ventral funiculus, as visualized by the accumulation of Hoechst-positive nuclei. Cell counts are expressed as percentage (ratio of cells expressing two markers divided by the number of cells expressing a single marker multiplied by 100) or density per square millimeter (number of cells expressing one or two markers divided by the area in square micrometers multiplied by 1,000,000). A minimum of three sections from three mice were analyzed and the average proportion or density of cells was determined per mouse. The average and SEM was calculated for each group using Microsoft Excel.

##### Statistics.

All statistics were performed using Prism. Data are represented as mean ± SEM. Significance was determined using either two-tailed Student's *t* tests or one-way ANOVA with Bonferroni test for *post hoc* analysis. Statistical significance is reported as *p ≤ 0.05, **p ≤ 0.01, ***p ≤ 0.001, ****p < 0.0001.

##### Mitochondrial labeling.

To quantify mitochondrial density, primary oligodendrocyte cells cultured for 10 d *in vitro* (DIV) or primary OPCs cultured for 14 DIV were treated with PBS (control), creatine (100 μm), or creatine and GPA (100 μm) for 24 h, after which mitochondria were labeled with MitoTracker Red (5 nm) for 30 min at 37°C. Mature oligodendrocytes and OPCs were identified via immunostaining for MBP and PDGFRα, respectively. Mitochondria outside the cell body were quantified using thresholded images in ImageJ. To measure mitochondrial membrane potential, purified oligodendrocyte lineage cells plated on a 96-well plate were differentiated until DIV 7 and treated with PBS or creatine (100 μm) for 24 h. Cells were incubated in 20 nm TMRE for 20 min and fluorescent intensity was measured using GloMax-Multi Detection System. As a control for TMRE sensitivity, cells were subsequently incubated with 20 μm FCCP, a known ionophore uncoupler of oxidative phosphorylation, for 20 min to confirm a reduction in TMRE signal.

##### Seahorse extracellular flux analysis.

O_2_ consumption rate of primary oligodendrocyte lineage cells was measured using an XF^e^96 Seahorse Bioscience Extracellular Flux Analyzer according to the manufacturer's protocol. Briefly, oligodendrocyte lineage cells were differentiated in defined medium and treated on DIV 3 with either creatine (100 μm) or PBS for 24 h. DIV 4 cells were washed in prewarmed assay medium for 1 h in a CO_2_-free 37°C incubator. The XF Cell Mito Stress Test Kit (Seahorse Bioscience, 103015–100) was used to quantify ATP production in *n* = 2 wells/condition after sequential injections of oligomycin, FCCP, and rotenone/antimycin A. Oligomycin, a complev IV inhibitor, was injected to differentiate ATP-linked respiration from proton leak. FCCP, an uncoupler of ATP synthesis, was next injected to measure maximal respiratory rate. Finally, rotenone/antimycin A, an inhibitor of complex III, was injected to measure all nonmitochondrial sources of oxygen consumption (Seahorse Bioscience).

##### Membrane expansion analysis.

To measure oligodendrocyte membrane expansion, purified oligodendrocyte lineage cells were differentiated until DIV 10 and treated with PBS (control), creatine (100 μm), or creatine and GPA (100 μm) for 24 h and immunostained for MBP. Fluorescent images were acquired using an EVOS Cell Imaging system and thresholded in ImageJ. Fractal dimensions of individual oligodendrocytes were quantified using the ImageJ Fractal Analysis plugin.

##### *TUNEL*.

TUNEL was performed using Click-iT TUNEL Alexa Fluor 594 Imaging Assay for microscopy and HCS according to the manufacturer's instructions (Invitrogen).

##### EdU labeling.

5-Ethynyl-2′-deoxyuridine (EdU) labeling was performed using the Click-iT EdU Alexa Fluor 594 Imaging Kit according to the manufacturer's instructions (Invitrogen). EdU was added to cell cultures at a final concentration of 25 μm beginning at the time of PBS or creatine treatment.

##### PI staining.

Cells were incubated with 10 μg/ml PI for 20 min at 37°C, washed once with prewarmed culture medium, and fixed for immunostaining.

##### RT-PCR.

Reverse transcription PCR (RT-PCR) was conducted on complementary DNA (cDNA) obtained from cultures of MACS-purified oligodendrocyte lineage cells. The following mouse primer sequences were synthesized by MWG Operon: *Agat* forward (5′-TCA CGC TTC TTT GAG TAC CG-3′), *Agat* reverse (5′-TCA GTC GTC ACG AAC TTT CC-3′), *Gamt* forward (5′-TGG CAC ACT CAC CAG TTC A-3′), *Gamt* reverse (5′-GAC TGC CGC TAC TAT GCC TT-3′), *CrT* forward (5′-TCC TGG CAC TCA TCA ACA G-3′), and *CrT* reverse (5′-ATG AAG CCC TCC ACA CCT AC-3′).

## Results

### Oligodendrocytes express components of the creatine biosynthesis and transport pathway

**Figure 1.**
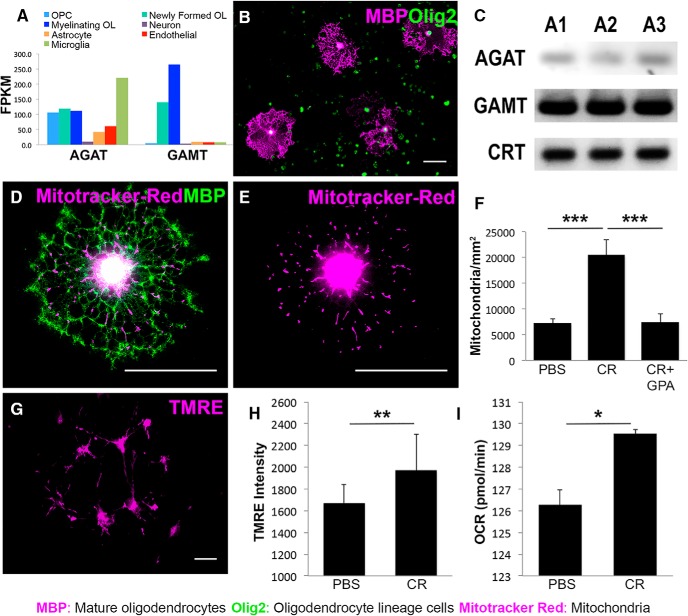
Creatine increases oligodendrocyte mitochondria density, membrane potential, and ATP production. ***A***, Graphical representation of cell-specific *Agat* and *Gamt* RNA levels obtained from a publicly available RNA-sequencing transcriptome database (http://web.stanford.edu/group/barres_lab/). FPKM represents fragments per kilobase of transcript sequence per million mapped fragments (Zhang et al., 2014). ***B***, DIV 10 oligodendrocyte lineage cells cultured via MACS. OPCs are Olig2^+^ (green) and mature oligodendrocytes are double positive for Olig2^+^ and MBP^+^ (magenta) (20×) ***C***, MACS-purified oligodendrocytes from *n* = 3 mice (A1–A3) express transcripts for the creatine biosynthetic enzymes, *Agat* and *Gamt*, and the creatine transporter, CrT, via RT-PCR. ***D***, MBP^+^ (green) oligodendrocyte with mitochondria (magenta) labeled with MitoTracker Red. ***E***, Same image shown in ***D*** displaying mitochondria (magenta) only. ***F***, Density of mitochondria per square millimeter counted in the processes of MBP^+^ oligodendrocytes in MACS-purified oligodendrocyte lineage cell cultures expanded until DIV 7 and differentiated until DIV 9, when they were treated with PBS, 100 μm creatine (CR), or CR + 100 μm GPA for 24 h. *n* = 12 cells/condition; one-way ANOVA with Bonferroni *post hoc* test. ***G***, Live-cell TMRE (magenta) image showing labeled mitochondria in living oligodendrocytes. ***H***, Quantification of average fluorescent intensity of TMRE signal from oligodendrocyte lineage cell cultures expanded for 24 h and differentiated until DIV 3, when they were treated with PBS or CR for 24 h. *n* = 2 wells/condition; Student's *t* test. ***I***, Results of Seahorse extracellular flux analysis showing average oxygen consumption rate (OCR) in picomoles per minute during ATP production in oligodendrocyte lineage cell cultures expanded for 24 h and differentiated until DIV 3, when they were treated with PBS or CR for 24 h. *n* = 2 wells/condition; Student's *t* test. Data are represented as mean ± SEM. Scale bars, 50 μm. Brightness and contrast were adjusted for visualization. *p < 0.05, **p < 0.01, ***p < 0.001, ****p < 0.0001.

Creatine synthesis occurs in a two-step reaction; l-arginine:glycine amidinotransferase (AGAT/GATM) first converts arginine and glycine into guanidinoacetate (GAA), which is then converted into creatine by guanidinoacetate-methyltransferase (GAMT). Oligodendrocytes express both AGAT and GAMT (Tachikawa et al., 2004; Braissant et al., 2010; Takasaki et al., 2010). To investigate how oligodendrocyte expression of these enzymes compares with that of other CNS cells, we searched for *Agat* (also known as *Gatm*) and *Gamt* in a publicly available RNA-sequencing transcriptome database of the mouse cerebral cortex (http://web.stanford.edu/group/barres_lab/brain_rnaseq.html; Zhang et al., 2014) and graphed the resulting FPKM (fragments per kilobase of transcript sequence per million mapped fragments) values (Fig. 1*A*). Astrocytes, neurons, OPCs, newly formed oligodendrocytes, myelinating oligodendrocytes, microglia, and endothelial cells all express *Agat*. However, newly formed and myelinating oligodendrocytes express 29× more *Gamt* than all other CNS cells combined (Zhang et al., 2014), indicating that mature oligodendrocytes have a preferentially high capacity for endogenous creatine synthesis in the CNS. To address the function of creatine in oligodendrocytes, we established cultures of purified primary mouse oligodendrocytes by isolating O4^+^ oligodendrocyte lineage cells from early postnatal mice using MACS. The resulting cultures contain 94% Olig2^+^ cells, of which 7% differentiate into mature, MBP-positive oligodendrocytes after 3 d in defined medium (Dincman et al., 2012) (Fig. 1*B*). RT-PCR conducted on cDNA isolated from these cultures confirmed the expression of *Agat*, *Gamt*, and creatine transporter 1 (*CrT*) (Fig. 1*C*), indicating that oligodendrocytes express all components of the creatine biosynthetic and transport pathway.

### Creatine increases oligodendrocyte mitochondria density, membrane potential, and ATP production

Creatine addition has been shown to increase mitochondrial membrane potential and transport in *Xenopus* spinal neurons (Lee and Peng, 2008) and ATP production in hippocampal neurons (Li et al., 2004) and muscle (Walsh et al., 2001). To determine whether creatine also increases ATP production in oligodendrocyte mitochondria, MACS-purified oligodendrocyte lineage cells were treated with 1× PBS, 100 μm creatine, or creatine and the competitive antagonist of CrT, guanidinopropionic acid (GPA; 100 μm) for 24 h. MitoTracker-Red was used to label mitochondria in mature oligodendrocytes, which were identified by expansive MBP staining (Fig. 1*D*,*E*). We found that creatine significantly increased the density of mitochondria in oligodendrocyte processes (*n* = 12 cells/condition; *p* = 0.0002). Moreover, this effect was completely abrogated by cotreatment with GPA (Fig. 1*F*; *n* = 12 cells/condition; *p* = 0.0002). MitoTracker-Red was also used to quantify mitochondria density in PDGFRα^+^ OPCs harvested by mixed glia shake off (Daniele et al., 2014) and treated with PBS, creatine, and creatine + GPA for 24 h. Mitochondria within a 50 μm region of a primary OPC process were quantified for analysis. We found that, compared with PBS (0.100 mitochondria/μm), mitochondria density was not significantly different in creatine (0.106 mitochondria/μm) or creatine + GPA (0.08 mitochondria/μm)-treated OPC processes (data not shown), indicating that creatine affects mature oligodendrocyte mitochondria density specifically within the lineage. Because density is linked to mitochondrial activity (Morris and Hollenbeck, 1993), we next examined the effect of creatine on mitochondrial activity in oligodendrocytes. Mitochondria within primary oligodendrocyte lineage cells were labeled with TMRE (Fig. 1*G*), a cationic dye that accumulates in mitochondria relative to their membrane potential (ψm) and serves as a proxy for ATP production such that its fluorescent intensity increases as ATP is produced via oxidative phosphorylation (Perry et al., 2011). We found that oligodendrocyte lineage cells treated with creatine for 24 h had significantly higher TMRE intensity compared with cells treated with PBS (Fig. 1*H*; *n* = 2 wells/condition; *p* = 0.0016), suggesting that the creatine treatment increases oligodendrocyte mitochondrial activity. We confirmed this finding using Seahorse extracellular flux analysis, which measures oxygen consumption as an indicator of mitochondrial respiration. We found that creatine-treated oligodendrocyte lineage cells displayed significantly increased ATP production compared with controls (Fig. 1*I*; *n* = 2 wells/condition; *p* = 0.0454). Together, these findings suggest that creatine enhances mitochondrial function directly in primary oligodendrocytes.

### Creatine treatment enhances oligodendrocyte lineage cell survival, but does not affect oligodendrocyte membrane expansion or differentiation

**Figure 2.**
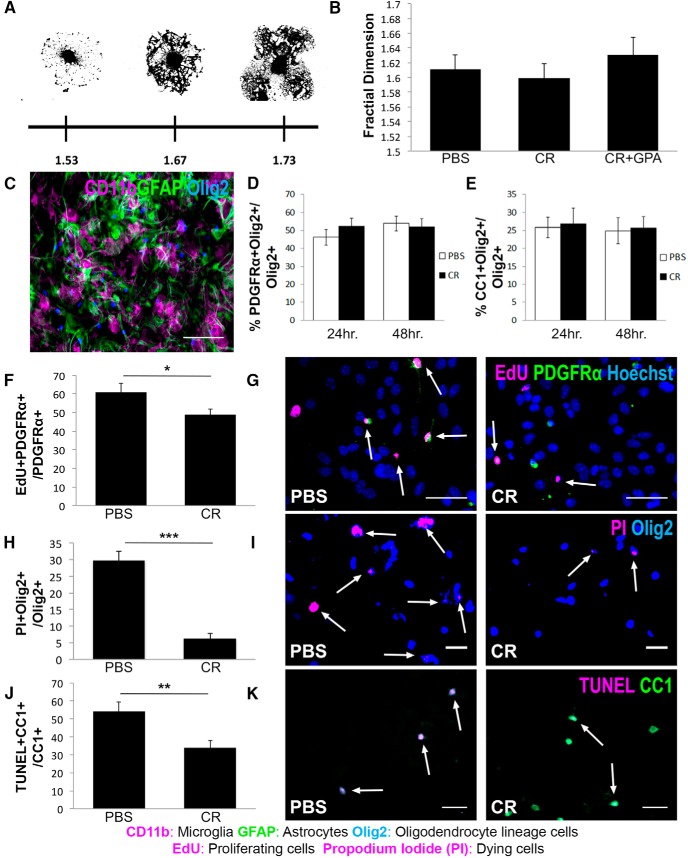
Creatine treatment enhances oligodendrocyte lineage cell survival, but does not affect oligodendrocyte membrane expansion or differentiation. ***A***, Representative images of MACS-cultured primary oligodendrocytes at different stages of membrane expansion and their associated fractal dimension values. ***B***, Fractal dimensions of primary oligodendrocytes treated with PBS, 100 μm creatine (CR), or CR + 100 μm GPA for 24 h. ***C***, Representative image of primary mouse mixed glia cultures containing microglia (CD11b^+^; magenta), astrocytes (GFAP^+^; green), and oligodendrocyte lineage cells (Olig2^+^; blue). Scale bar, 100 μm. ***D***, Quantification of immunostaining showing the percentage of OPCs (PDGFRα^+^Olig2^+^) of total oligodendrocyte lineage cells (Olig2^+^) after 24 or 48 h of treatment with PBS or CR. *n* = 9 images/condition; Student's *t* test. ***E***, Quantification of immunostaining showing the percentage of mature oligodendrocytes (CC1^+^Olig2^+^) of total oligodendrocyte lineage cells (Olig2^+^) after 24 or 48 h ot treatment with PBS or CR. *n* = 9 images/condition; Student's *t* test. ***F***, Quantification of immunostaining showing the percentage of proliferating OPCs (Edu^+^PDGFRα^+^) out of total OPCs (PDGFRα^+^) after 48 h of treatment with PBS or CR. *n* = 10 images/condition; Student's *t* test. ***G***, Representative images of EdU (magenta) immunostaining showing proliferating OPCs (PDGFRα^+^, green) in mixed glia cultures after 48 h of treatment with PBS or CR. White arrows indicate Edu^+^PDGFRα^+^ cells. Scale bars, 50 μm. ***H***, Quantification of immunostaining showing the percentage of dying oligodendrocyte lineage cells (PI^+^Olig2^+^) of total oligodendrocyte lineage cells (Olig2^+^) after 48 h of treatment with PBS or CR. *n* = 5 images/condition; Student's *t* test. ***I***, Representative images of PI (magenta) immunostaining showing dying oligodendrocyte lineage cells (Olig2^+^, blue) in mixed glia cultures after 48 h of treatment with PBS or CR. White arrows indicate PI^+^Olig2^+^ cells. Data are represented as mean ± SEM. Scale bars, 50 μm. ***J***, Quantification of TUNEL assay and immunostaining showing the percentage of dying oligodendrocytes (TUNEL^+^CC1^+^) of total oligodendrocytes (CC1^+^) after 48 h of treatment with PBS or CR. *n* = 12 images/condition; Student's *t* test. ***K***, Representative images of TUNEL (magenta) assay and immunostaining of dying oligodendrocytes (CC1^+^, green) in mixed glia cultures after 48 h of treatment with PBS or CR. White arrows indicate TUNEL^+^CC1^+^ cells. *p < 0.05, **p < 0.01, ***p < 0.001, ****p < 0.0001.

Myelination is a highly energy-demanding process, requiring an estimated 3.30 × 10^23^ ATP molecules/g of myelin synthesized (Harris and Attwell, 2012). To determine whether increased ATP production in creatine-treated oligodendrocytes stimulates membrane expansion, MACS-purified oligodendrocyte lineage cells were treated with PBS (control), creatine (creatine), or creatine + GPA for 24 h. Expansion of MBP-positive membranes was measured using fractal analysis, a quantitative measurement of morphological complexity that has been used previously to assess differentiation of oligodendrocyte lineage cells *in vitro* (Behar, 2001). We found that the fractal dimension of all oligodendrocytes was in the range of those previously reported (Fig. 2*A*). However, the average fractal dimension of oligodendrocytes was not significantly different across treatment groups compared with control (Fig. 2*B*), suggesting that creatine does not affect oligodendrocyte membrane expansion *in vitro*.

To test whether creatine addition stimulates differentiation of OPCs into oligodendrocytes, we quantified the proportions of OPCs and mature oligodendrocytes in primary mixed glia cultured in serum-free medium with PBS or creatine for 24 or 48 h (Fig. 2*C*). Creatine treatment did not affect the proportions of OPCs (Fig. 2*D*) or oligodendrocytes (Fig. 2*E*) at either time point (*n* = 9 images/condition), suggesting that creatine does not stimulate differentiation of OPCs into oligodendrocytes. Because no difference was observed in the proportions of oligodendrocyte lineage cells after creatine treatment, we investigated whether creatine affected the homeostatic turnover of oligodendrocyte lineage cells (Hughes et al., 2013) by assessing the overall proportion of cells undergoing proliferation and cell death. Primary mixed glia were treated with PBS or creatine for 48 h in the presence of the thymidine analog EdU to label proliferating cells engaged in DNA synthesis. Quantification of cells immunostained for EdU and PDGFRα revealed that creatine treatment resulted in a significant reduction in the proportion of proliferating oligodendrocyte precursor cells (Fig. 2*F*,*G*; *n* = 10 images/condition; *p* = 0.0439). To determine whether creatine affects oligodendrocyte lineage cell death, mixed glia treated with PBS or creatine for 48 h were incubated with PI, a membrane-impermanent DNA-intercalating agent used for the identification of dying cells. We found that creatine treatment reduced the proportion of dying oligodendrocyte lineage cells significantly (Fig. 2*H*,*I*; *n* = 5 images/condition; *p* = 0.0002), suggesting that creatine promotes their survival. TUNEL analysis for the detection of fragmented DNA in dying CC1^+^ oligodendrocytes further demonstrated that creatine improved the survival of mature oligodendrocytes significantly (Fig. 2*J*,*K*; *n* = 12 images/condition; *p* = 0.0070). To investigate the direct effect of creatine on oligodendrocyte survival, MACS-purified oligodendrocyte lineage cells were treated with PBS or creatine for 24 h. Creatine-treated cultures exhibited a smaller proportion of dying oligodendrocytes (% PI^+^MBP^+^/MBP^+^) compared with PBS-treated cultures (16.37% vs 45.27%; *n* = 6 images/condition; *p* = 0.0273, Student's *t* test; data not shown), demonstrating a direct prosurvival effect of creatine on mature oligodendrocytes.

### Creatine promotes oligodendrocyte cell survival after inflammatory insult

**Figure 3.**
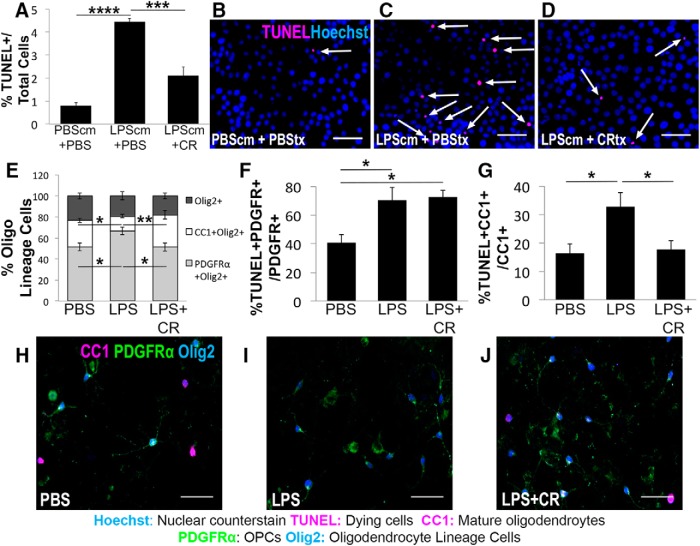
Creatine promotes oligodendrocyte cell survival following inflammatory insult. ***A***, Quantification of TUNEL assay and immunostaining showing the percentage of dying OLN cells (TUNEL^+^Hoechst^+^) of total cells (Hoechst^+^) after treatment. OLN-93 cells were treated with PBS or 100 μm creatine (CR) for 24 h while simultaneously being exposed to conditioned medium (cm) from RAW cells treated with either PBS (PBScm) or 1 μg/ml LPS (LPScm) the day prior. *n* = 10 images/condition; one-way ANOVA with Bonferroni *post hoc* test. ***B***–***D***, TUNEL assay (magenta) depicting dying OLN-93 cells (TUNEL^+^Hoechst^+^) in cultures treated with PBScm + PBS (***B***), LPScm + PBS (***C***), or LPScm + CR (***D***). ***E***, Quantification of immunostaining showing the percentage of total Olig2^+^ cells (dark gray) represented by OPCs (PDGFRα^+^Olig2^+^; light gray) and mature oligodendrocytes (CC1^+^Olig2^+^; white) after 24 h of treatment with PBS, LPS, or LPS + CR. *n* = 10 images/condition; one-way ANOVA with Bonferroni *post hoc* test. ***F***, Quantification of the percentage of dying OPCs (TUNEL^+^PDGFRα^+^) out of total OPCs (PDGFRα^+^) after 24 h of treatment with PBS, LPS, or LPS+CR. *n* = 10 images/condition; one-way ANOVA with Bonferroni *post hoc* test. ***G***, Quantification of the percentage of dying oligodendrocytes (TUNEL^+^CC1^+^) of total oligodendrocytes (CC1^+^) after 24 h of treatment with PBS, LPS, or LPS + CR. *n* = 10 images/condition; one-way ANOVA with Bonferroni *post hoc* test. ***H***–***J***, Immunostaining for mature oligodendrocytes (CC1^+^Olig2^+^) and OPCs (PDGFRα^+^Olig2^+^) in primary mouse mixed glial cultures treated with PBS (***H***), LPS (***I***), or LPS + CR (***J***) for 24 h. Data are represented as mean ± SEM. Scale bars, 50 μm. Brightness and contrast were adjusted for visualization. *p < 0.05, **p < 0.01, ***p < 0.001, ****p < 0.0001.

The prosurvival effect of creatine (Fig. 2*H–K*) prompted us to test whether creatine also promotes oligodendrocyte survival under inflammatory conditions. We first established a robust assay of inflammation-mediated oligodendrocyte cell death using the rodent oligodendroglia cell line OLN-93 (Richter-Landsberg and Heinrich, 1996). To mimic a proinflammatory environment, OLN-93 cells were treated with conditioned media taken from RAW 264.7 macrophage/monocyte(s) treated with PBS or LPS. LPS is a potent activator of Toll-like 4 receptor (TLR4) that stimulates macrophages/monocytes to release proinflammatory cytokines and is a known activator of oligodendrocyte cell death in mixed cultures (Li et al., 2005). Cell death analysis by TUNEL assay showed that the addition of conditioned medium from LPS-activated macrophages to OLN-93 cells increased cell death significantly compared with the control conditioned medium (Fig. 3*A–D*; *n* = 10 images/condition; *p* < 0.0001). Moreover, the addition of creatine with the LPS-conditioned medium resulted in a dramatic reduction of OLN-93 cell death compared with PBS addition (Fig. 3*A*,*D; n* = 10 images/condition; *p* = 0.0008), suggesting that creatine has a direct prosurvival effect on oligodendrocytes. We next examined the prosurvival effect of creatine on primary oligodendrocytes by treating mixed glia cultures with PBS, LPS, or LPS + creatine, followed by coimmunostaining analysis with antibodies against PDGFRα, CC1, and Olig2. Compared with PBS, LPS treatment significantly reduced the proportion of CC1^+^Olig2^+^ mature oligodendrocytes (Fig. 3*E*, white bars; *n* = 10 images/condition; *p* = 0.0258). This effect was abrogated by cotreatment with creatine (Fig. 3*E*, white bars; *n* = 10 images/condition; *p* = 0.0006). As demonstrated previously (Li et al., 2008; Skripuletz et al., 2011), LPS treatment resulted in a higher proportion of PDGFRα^+^Olig2^+^ OPCs compared with PBS (Fig. 3*E*, light gray bars; *n* = 10 images/condition; *p* = 0.019), an effect that was abrogated by cotreatment with creatine (Fig. 3*E*, light gray bars; *n* = 10 images/condition; *p* = 0.017). Additionally, whereas LPS increased apoptosis of both OPCs (Fig. 3*F*, *n* = 8 images/condition; *p* = 0.016) and oligodendrocytes (Fig. 3*G*; *n* = 10 images/condition; *p* = 0.0482), cotreatment with creatine specifically ameliorated apoptosis of oligodendrocytes (Fig. 3*G*; *n* = 10 images/condition; *p* = 0.0179), but had no effect on OPC death, which remained significantly elevated compared with PBS (Fig. 3*F*; *n* = 10 images/condition; *p* = 0.010). The simultaneous increase in OPC proportion and apoptosis is mediated by increased OPC proliferation under LPS treatment (*n* = 10 images/condition; *p* = 0.023, Student's *t* test; data not shown), suggesting that the OPC response is largely homeostatic. Overall, these results further suggest that creatine promotes oligodendrocyte survival under proinflammatory conditions.

### Reduced oligodendrocyte density in *Gamt*-deficient focal demyelinating lesions can be rescued by creatine injection

We next investigated whether creatine deficiency *in vivo* impairs oligodendrocyte viability by analyzing mice lacking the creatine-synthesizing enzyme GAMT (*Gamt*^−/−^). These mice are completely deficient in endogenously synthesized creatine, but do not display the severe learning deficits characteristic of human creatine deficiency (Schmidt et al., 2004). We selected this model to ensure absence of peripherally synthesized creatine in the CNS (Schmidt et al., 2004; Torremans et al., 2005) and because oligodendrocytes are the only CNS cell type expressing high levels of *Gamt* (Zhang et al., 2014; Fig. 1*A*). Western blot analysis demonstrates that GAMT protein is undetectable in cortical and cerebellar protein lysates of *Gamt*^−/−^ mice. Moreover, we found that *Gamt*^−/−^ mice had normal expression of oligodendrocyte-specific proteins (Olig2, PLP, and MBP) at P30. Total levels of the mitochondrial-specific proteins Tom20 and VDAC were also not different between *Gamt*^+/+^ and *Gamt*^−/−^ mice (data not shown).

**Figure 4.**
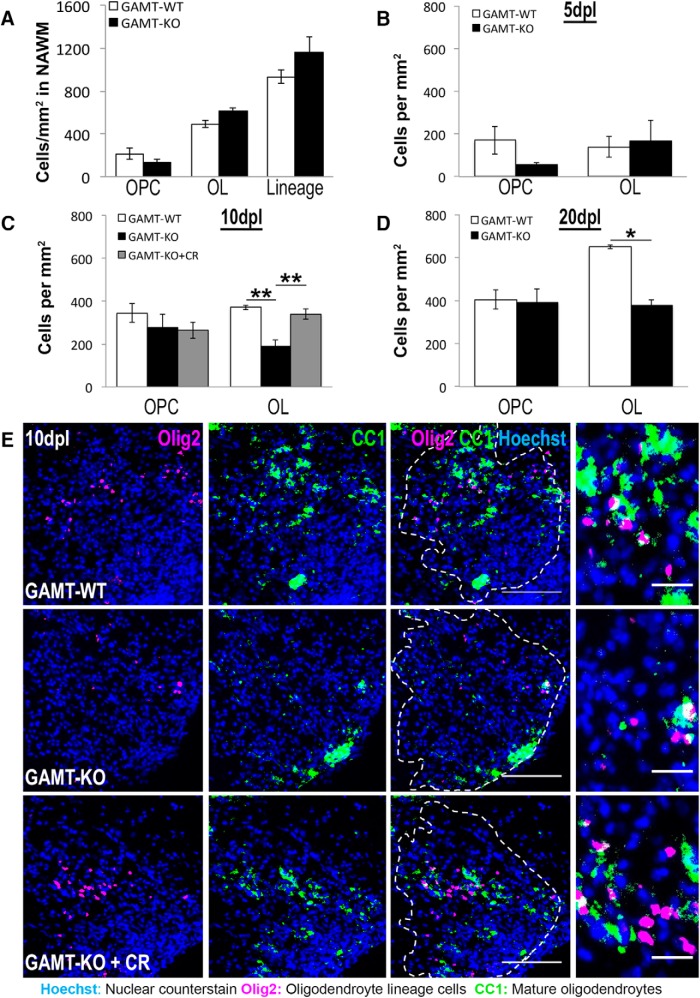
Reduced oligodendrocyte density in *Gamt*-deficient focal demyelinating lesions can be rescued by creatine injection. ***A***, Quantification of immunostainings for OPCs (Nkx2.2^+^Olig2^+^), oligodendrocytes (OL; CC1^+^Olig2^+^), and total oligodendrocyte lineage cells (Lineage; Olig2^+^) per square millimeter in NAWM in *Gamt*^+/+^ (GAMT-WT) and *Gamt*^−/−^ (GAMT-KO) mice. ***B***–***D***, Quantification of immunostainings for OPCs and OLs per square millimeter at 5 (***B***), 10 (***C***), and 20dpl (***D***) in GAMT-WT, GAMT-KO, and *Gamt*^−/−^ mice coinjected with 25 ng of creatine (GAMT-KO + CR; 10 dpl only). *n* = 3 mice/condition; one-way ANOVA with Bonferroni *post hoc* test performed in ***C***, Student's *t* test performed in ***D***. ***E***, Representative immunostainings of mature oligodendrocytes double positive for Olig2 (magenta) and CC1 (green) at 10 dpl. Data are represented as mean ± SEM. Long scale bars, 100 μm; short scale bars, 25 μm. Brightness and contrast were adjusted for visualization. *p < 0.05, **p < 0.01.

**Figure 5.**
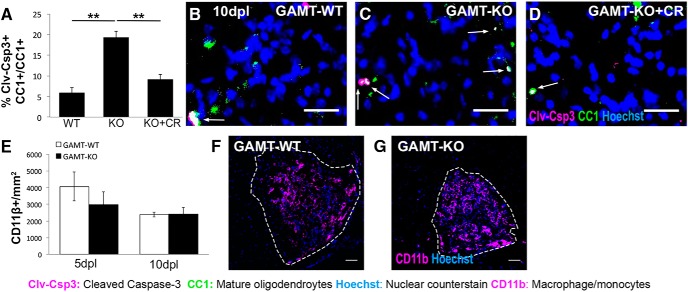
*Gamt*-deficient oligodendrocytes exhibit reduced survival after focal spinal cord demyelination. ***A***, Quantification of immunostaining showing the proportion of oligodendrocytes positive for cleaved caspase-3 (Clv-Csp3^+^CC1^+^) of total oligodendrocytes (CC1^+^) at 10 dpl in *Gamt*^+/+^ (GAMT-WT), *Gamt*^−/−^ (GAMT-KO), and *Gamt*^−/−^ mice treated with 25 ng of creatine (GAMT-KO + CR). *n* = 3 mice/condition; one-way ANOVA with Bonferroni *post hoc* test. ***B***–***D***, Representative immunostainings of dying oligodendrocytes in (GAMT-WT) (***B***), GAMT-KO (***C***), and GAMT-KO + CR (***D***) lesions at 10 dpl. White arrows indicate cells double positive for Clv-Csp3 and CC1. Scale bars, 25 μm. ***E***, Quantification of immunostaining for CD11b^+^ cells per square millimeter in GAMT-WT and GAMT-KO lesions at 5 and 10 dpl. *n* = 3 mice/condition. ***F***, ***G***, Representative immunostainings of macrophages/microglia (CD11b^+^; magenta) in GAMT-WT (***F***) and GAMT-KO (***G***) lesions at 5 dpl. Scale bars, 50 μm. Data are represented as mean ± SEM. Brightness and contrast were adjusted for visualization. *p < 0.05, **p < 0.01.

To investigate whether creatine plays a role in oligodendrocyte survival during remyelination *in vivo*, we analyzed a previously published remyelination transcriptome from the rat CNS (Huang et al., 2011). Similar to other highly expressed oligodendrocyte genes, including myelin oligodendrocyte glycoprotein (*Mog*) and myelin basic protein (*Mbp*), *Gamt* was significantly differentially upregulated during oligodendrocyte differentiation and remyelination at 14 and 28 d post lesion (dpl), respectively (*p* = 0.0019; Huang et al., 2011). Considering the time course of this upregulation, we hypothesized that *Gamt*-deficient oligodendrocytes would have impaired viability during remyelination. To test this, we used a mouse model of focal demyelination in which the toxin lysolecithin is microinjected into the mouse spinal cord white matter. This model was selected due to the known lesion location and well documented time course in which cell death and inflammation, oligodendrocyte differentiation, and remyelination occur at ∼5, 10, and 20 dpl, respectively (Pavelko et al., 1998; Bieber et al., 2003; Arnett et al., 2004). Focal spinal cord lesions were conducted on 9- to 12-week-old wild-type and *Gamt*^−/−^ mice and lesions were identified by focal accumulation of Hoechst-positive nuclei, thought to represent an influx of inflammatory cells to the injury site. *Gamt* deficiency did not affect OPC (Nkx2.2^+^Olig2^+^), oligodendrocyte (OL; CC1^+^Olig2^+^), or total oligodendrocyte lineage cell (Lineage; Olig2^+^) density in NAWM adjacent to lesions (Fig. 4*A*). Although *Gamt* deficiency had no effect on the recruitment of Nkx2.2^+^Olig2^+^ OPCs to the lesions across all three time points examined (Fig. 4*B–D*), significantly fewer CC1^+^Olig2^+^ oligodendrocytes were present in *Gamt*^−/−^ lesions at 10 dpl (Fig. 4*C*,*E*; *n* = 3 mice/condition; *p* = 0.003) and 20 dpl (Fig. 4*D*; *n* = 3 mice/condition; *p* = 0.0185), suggesting that *Gamt* deficiency impaired oligodendrocyte differentiation or reduced the survival of newly regenerated oligodendrocytes. Moreover, coinjection of 25 ng of creatine with lysolecithin into *Gamt*^−/−^ animals at the time of surgery (GAMT-KO + creatine) led to significantly more CC1^+^Olig2^+^ oligodendrocytes in the lesion at 10 dpl compared with *Gamt*^−/−^ animals coinjected with PBS (Fig. 4*C*,*E*; *n* = 3 mice/condition; *p* = 0.008), suggesting that creatine treatment is able to rescue oligodendrocyte density in *Gamt*-deficient lesions. The robust prosurvival effect of creatine *in vitro* (Figs. 2, 3) led us to hypothesize that the reduction of oligodendrocytes in *Gamt*^−/−^ lesions may be the result of impaired oligodendrocyte viability. To quantify oligodendrocyte cell death in lesions, coimmunostaining analysis for CC1 and cleaved caspase-3 (Clv-Csp3), a critical executioner protein involved in oligodendrocyte cell death (Casaccia-Bonnefil, 2000), was performed. Although the number of Clv-Csp3^+^CC1^+^ oligodendrocytes was not different between groups at 5 dpl (data not shown), it was significantly increased in *Gamt*^−/−^ lesions at 10 dpl (Fig. 5*A–C*; *n* = 3 mice/condition; *p* = 0.001). Compared to coinjection with PBS, coinjection with creatine reduced the number of Clv-Csp3^+^CC1^+^ oligodendrocytes in *Gamt*^−/−^ lesions by 47% (Fig. 5*A*,*D*; *n* = 3 mice/condition; *p* = 0.004). Therefore, despite regenerating from OPCs, oligodendrocytes within *Gamt*-deficient lesions are unable to survive in the absence of creatine. To investigate whether changes in inflammation contributed to altered oligodendrocyte viability in lesions, we quantified the density of CD11b^+^ macrophages/microglial cells at 5 and 10 dpl and found no differences between wild-type and *Gamt*^−/−^ mice at 5 or 10 dpl (Fig. 5*E–G*), suggesting that GAMT does not affect macrophage/microglia number in lesions and is required specifically for oligodendrocyte viability.

### Creatine administration increases oligodendrocyte density after focal spinal cord demyelination

**Figure 6.**
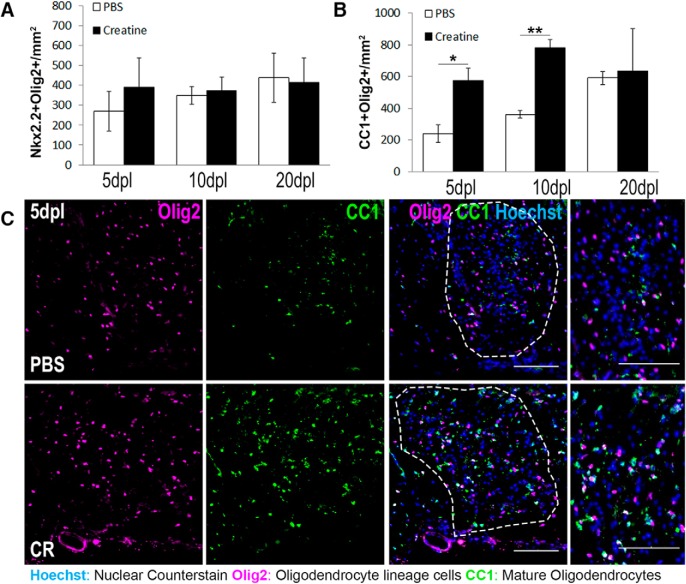
Creatine administration enhances oligodendrocyte restoration after focal spinal cord demyelination. ***A***, ***B***, Quantification of immunostaining for Nkx2.2^+^Olig2^+^ OPCS per square millimeter (***A***) and CC1^+^Olig2^+^ oligodendrocytes per square millimeter (***B***) at 5, 10, and 20 dpl in mice treated with PBS or 25 ng of creatine (CR). *n* = 3 mice/condition; Student's *t* test. ***C***, Representative immunostaining of mature oligodendrocytes double positive for Olig2 (magenta) and CC1 (green) in PBS and CR lesions at 5dpl. Data are represented as mean ± SEM. Scale bars, 100 μm. Brightness and contrast were adjusted for visualization. *n* = 3 mice/condition; *p < 0.05, **p < 0.01, ***p < 0.001, ****p < 0.0001.

**Figure 7.**
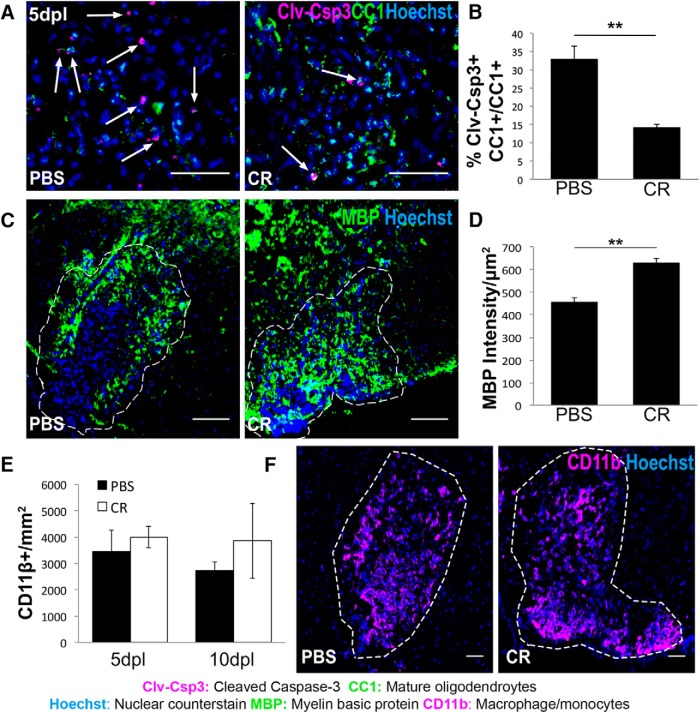
Creatine administration enhances oligodendrocyte survival after focal spinal cord demyelination. ***A***, Representative immunostainings of dying oligodendrocytes in mice treated with PBS or 25 ng of creatine (CR) at 5dpl. White arrows indicate cells double positive for Clv-Csp3 and CC1. Scale bars, 50 μm. ***B***, Quantification of immunostaining showing the proportion of oligodendrocytes positive for cleaved caspase-3 (Clv-Csp3^+^CC1^+^) of total oligodendrocytes (CC1^+^) in PBS and CR lesions at 5dpl. *n* = 2 mice/condition; Student's *t* test. ***C***, Representative immunostainings of MBP in PBS-treated and creatine-treated (CR) lesions at 20 dpl. Scale bars, 100 μm. ***D***, Quantification of immunostaining showing average MBP intensity per square micrometer in PBS and CR lesions at 20dpl. *n* = 3 mice/condition; Student's *t* test. ***E***, Quantification of immunostaining for CD11b^+^ cells/mm^2^ in PBS and CR lesions at 5 and 10 dpl. *n* = 3 mice/condition; Student's *t* test. ***F***, Representative immunostainings of macrophages/microglia (CD11b^+^; magenta) in PBS and CR lesions at 5dpl. Data are represented as mean ± SEM. Scale bars, 50 μm. Brightness and contrast were adjusted for visualization. *p < 0.05, **p < 0.01, ***p < 0.001, ****p < 0.0001.

Creatine has proven safe and well tolerated in clinical trials (Malin et al., 2008; Rosenfeld et al., 2008), but its effect on oligodendrocyte survival in MS remains unknown. To determine whether exogenous creatine administration promotes oligodendrocyte survival in wild-type CNS lesions, spinal cords of WT mice were coinjected with lysolecithin and either PBS or 25 ng of creatine. We found that creatine had no effect on the number of Nxk2.2^+^Olig2^+^ OPCs at any of the postlesion time points examined (Fig. 6*A*; *n* = 3 mice/condition), but increased the density of CC1^+^Olig2^+^ oligodendrocytes significantly at both 5 and 10 dpl (Fig. 6*B*,*C*; *n* = 3 mice/condition; *p* = 0.0254, *p* = 0.0022). The observation that creatine-treated mice exhibited increased oligodendrocyte density suggests that creatine may promote the survival of oligodendrocytes within the lesion. Indeed, we observed that creatine-treated mice had significantly fewer Clv-Csp3^+^ oligodendrocytes at 5 dpl compared with PBS-treated mice (Fig. 7*A*,*B*; *n* = 3 mice/condition; *p* = 0.0051), demonstrating a role for creatine in the inhibition of caspase-dependent oligodendrocyte apoptosis. Moreover, we detected increased MBP staining in lesions at 20 dpl in creatine-treated mice compared with control, suggesting that creatine-mediated oligodendrocyte survival enhanced CNS remyelination (Fig. 7*C*,*D*; *n* = 3 mice/condition; *p* = 0.0019). Because creatine did not affect the density of inflammatory macrophages/microglia at 5 or 10 dpl (Fig. 7*E*,*F*), changes in inflammation are unlikely to mediate the beneficial effect of creatine. This result suggests that creatine administration can enhance oligodendrocyte survival directly in the CNS.

## Discussion

The CNS uses 20% of the body's energy (Mergenthaler et al., 2013), with the human cortex alone requiring ∼3 × 10^23^ ATP/s/m^3^ (Howarth et al., 2012). Creatine is thought to play a critical role in meeting this energy demand by allowing for rapid ATP regeneration in the cytoplasm (Wyss and Kaddurah-Daouk, 2000). Within the CNS, oligodendrocytes have a preferentially high capacity for creatine synthesis (Braissant et al., 2001; Tachikawa et al., 2004; Cahoy et al., 2008; Zhang et al., 2014), suggesting that they may be a major source of creatine in the brain. Therefore, we investigated the role of creatine in oligodendrocytes using both *in vitro* and *in vivo* approaches.

In purified oligodendrocyte lineage cells, creatine increased oligodendrocyte mitochondrial density, membrane potential, and ATP production directly. We found that enhanced mitochondrial function in creatine-treated oligodendrocytes did not increase membrane expansion or differentiation, but rather promoted oligodendrocyte survival. Creatine treatment of primary mixed glia cultures for 48 h reduced oligodendrocyte cell death significantly. Further, OPC proliferation was also reduced in creatine-treated cultures. Despite these underlying changes, no differences were observed in the overall proportions of OPCs and oligodendrocytes after either 24 or 48 h of treatment. Because we did not quantify O4^+^MBP^−^ preoligodendrocytes directly, we cannot rule out the possibility that creatine may have affected the proportion of this intermediate population. However, at the experimental end point, O4^+^ highly overlapped with MBP^+^ in our cultures, making robust quantification of this population difficult. Inclusion of additional time points in the 24–48 h treatment window could address this question in future studies. The simultaneous changes in oligodendrocyte death and OPC proliferation suggest an active mechanism for maintaining homeostatic density of oligodendrocytes reminiscent to that recently described *in vivo* (Hughes et al., 2013).

In contrast, acute (24 h) LPS-mediated inflammatory injury altered the proportions of oligodendrocyte lineage cells significantly. LPS treatment reduced the proportion of oligodendrocytes and concomitantly increased oligodendrocyte apoptosis, both of which were returned to control levels by cotreatment with creatine. As reported previously, we found that OPCs exhibit both increased apoptosis and proliferation in response to LPS treatment (Li et al., 2008; Skripuletz et al., 2011). However, creatine did not affect either of these parameters significantly, indicating that changes in OPC proportion are a reflection of oligodendrocyte loss rather than a direct effect of creatine on OPCs. It is interesting that creatine did not reduce the level of oligodendrocyte apoptosis below PBS in this experiment. This effect may be due to the acute timing of LPS treatment (24 h) rather than different underlying mechanisms of inflammatory and noninflammatory cell death *in vitro* because previous work suggests that oligodendrocytes undergo apoptosis both as a normal turnover response and as a result of cytokine- or glutamate-induced excitotoxicity in the presence of activated microglia (Barres et al., 1992; Matute et al., 2006; Aktas at al., 2006).

To determine whether creatine also affected oligodendrocyte survival *in vivo*, we conducted loss-of-function experiments by performing focal spinal cord demyelination on *Gamt*-deficient mice and quantifying the process of spontaneous oligodendrocyte regeneration. Lesions of *Gamt*-deficient mice contained fewer total oligodendrocytes and a higher proportion of cleaved caspase-3-positive oligodendrocytes compared with controls. Injection of creatine at the time of surgery rescued the detrimental effect of *Gamt* deficiency on oligodendrocyte apoptosis, suggesting that creatine is necessary for survival of newly generated oligodendrocytes. Oligodendrocyte lineage cell numbers were not different in the NAWM of *Gamt*-deficient mice, suggesting that survival of the oligodendrocyte lineage is either not affected during development or compensatory mechanisms allow for normalization of these densities over time.

Further supporting a role for creatine in the survival of newly generated oligodendrocytes, lesions treated with exogenous creatine at the time of injury contained more oligodendrocytes at 5 and 10 dpl due to a reduction in caspase-mediated oligodendrocyte cell death. Interestingly, by 20 dpl, PBS-treated lesions contain similar numbers of oligodendrocytes as those treated with creatine. Therefore, it appears that creatine serves to promote oligodendrocyte survival early in the injury time course, when inflammation remains high. Creatine treatment was also associated with elevated MBP expression, suggesting that creatine may enhance myelin synthesis or speed up the spontaneous process of myelin regeneration *in vivo*. Addition of exogenous creatine or loss of *Gamt* did not affect the recruitment of inflammatory cells, suggesting that creatine promotes oligodendrocyte viability directly.

**Figure 8.**
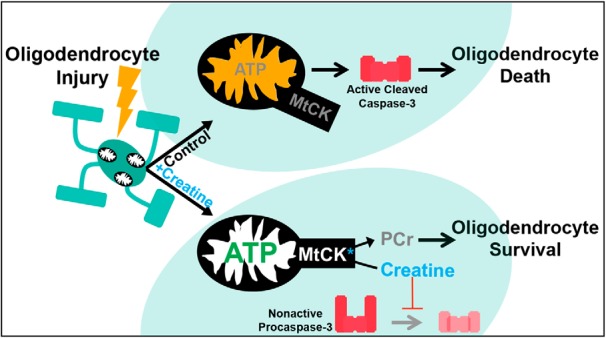
Hypothesized model for creatine-mediated protection of oligodendrocytes. Creatine treatment promotes oligodendrocyte restoration after demyelinating injury by inhibiting caspase-dependent oligodendrocyte death. Although the precise mechanism remains to be elucidated, it is hypothesized that creatine activation of MtCK may inhibit apoptotic initiation directly.

Despite the high expression of creatine-synthesizing enzymes in oligodendrocytes (Tachikawa et al., 2004; Braissant et al., 2010; Takasaki et al., 2010; Zhang et al., 2014), its physiological importance in these cells had not been investigated. Our results suggest a model in which creatine promotes the viability of newly generated oligodendrocytes by enhancing mitochondrial function (Fig. 8). Several mechanisms of creatine-mediated protection have been proposed. Creatine can serve as a direct antioxidant (Lawler et al., 2002) and reduces markers of oxidative stress in rodent models of neurological insult (Hosamani et al., 2010; Saraiva et al., 2012; Cunha et al., 2013; Rambo et al., 2013). Creatine also inhibits loss of mitochondrial membrane potential (Rambo et al., 2013), which has been shown to precede initiation of cellular apoptosis (Kroemer et al., 2007). In addition, creatine activation of MtCK directly inhibits the opening of the mitochondrial permeability transition pore, an early apoptotic event concomitant with cytochrome-c release (Beutner et al., 1996; O'Gorman et al., 1997; Beutner et al., 1998; Vyssokikh and Brdiczka, 2003; Schlattner et al., 2006). Our loss- and gain-of-function studies yielded complementary results, demonstrating that creatine increases oligodendrocyte density during CNS regeneration by inhibiting caspase-dependent cell death of mature oligodendrocytes. Although caspase-3 can be activated by both intrinsic (mitochondrial) and extrinsic (death receptor) pathways, our finding that creatine increases mitochondrial membrane potential and ATP production directly in oligodendrocyte lineage cells suggests that creatine protection is mediated by a mitochondrial-dependent mechanism. Future studies are necessary to address whether creatine activation of MtCK promotes oligodendrocyte viability.

Oligodendrocyte death plays a crucial role in the pathology of MS (Matute et al., 2006; Macchi et al., 2015). In demyelinated lesions, oligodendrocyte death may be achieved with or without complement activation (Lucchinetti et al., 2000; Barnett and Prineas, 2004). Moreover, oligodendrocytes can undergo either apoptosis or necrosis, depending on how mitochondrial function is affected (Casaccia-Bonnefil, 2000). Many oligodendrocytes appear to survive demyelination in chronic-stage MS, but are lost from lesioned areas gradually over time (Wolswijk, 2000), suggesting that their survival may be compromised under chronic inflammation. Preventing oligodendrocyte cell death in MS is particularly important considering that current therapies do not prevent transition into secondary-progressive disease (Huang and Franklin, 2012), in which chronic demyelination is thought to contribute to neurodegeneration (Jeffery and Blakemore, 1997; Kornek et al., 2000; Irvine and Blakemore, 2008). In addition, the past few decades have demonstrated novel roles for oligodendrocytes in maintaining neuronal health and integrity (Nave and Trapp, 2008; Fünfschilling et al., 2012; Lee et al., 2012), underscoring the importance of maintaining the oligodendrocyte-axon connection. Our work demonstrates that creatine promotes survival of oligodendrocytes under inflammatory conditions both *in vitro* and *in vivo*. It is therefore exciting to consider creatine as a potential treatment strategy for protecting oligodendrocytes in patients with MS. Dietary creatine supplementation has proven safe and well tolerated in clinical trials (Kahler and Fahey, 2003). It has also been demonstrated to improve brain performance (Rae et al., 2003) and protect neurons during oxygen deprivation (Turner et al., 2015). Therefore, future work is needed to elucidate whether dietary creatine supplementation or the administration of creatine analogs can protect oligodendrocytes in both the laboratory and clinical setting.

In summary, we have found a novel role for creatine in promoting oligodendrocyte viability. Our results suggest that creatine may be a potentially relevant therapeutic agent for promoting oligodendrocyte survival in MS.
